# Splenic Angiosarcoma Presenting With Jaundice, Ascites and Bone Marrow Fibrosis

**DOI:** 10.1080/13577140310001644814

**Published:** 2003

**Authors:** Kim Vaiphei, Virinder Singh, Subhash Varma

**Affiliations:** 1 Department of Histopathology Post Graduate Institute of Medical Education and Research No. 127/C, Sector-24/A Chandigarh Pin-160023 India; 2 Department of Hepatology Post Graduate Institute of Medical Education and Research Chandigarh India; 3 Department of Internal Medicine Post Graduate Institute of Medical Education and Research Chandigarh India

## Abstract

A middle aged chronic alcoholic presented with deep jaundice, markedly enlarged and tender spleen with leukoerythroblastic blood picture and bone marrow biopsy showing mild fibrosis. He was tested negative for HIV, hepatitis B and C viruses. Besides very high serum bilirubin, alkaline phosphatase was raised four times the normal value. Contrast enhanced CT showed enlarged spleen and liver with multiple heterogenous lesions in spleen and tiny hypo-dense lesions in liver. In hospital, he developed haemolytic uraemic syndrome and succumed to his illness. At autopsy spleen weighed 5200 gms and variegated in appearance due to large areas of necrosis and whitish tumour nodules. Histology revealed morphology of an angiosarcoma. Liver was also infiltrated by the tumour mainly in and around portal tract areas.


*Sarcoma*, September/December 2003, *VOL. 7, NO. 3/4, 183–184*

Taylor & Francis healthsciences

**CASE REPORT**

### Splenic angiosarcoma presenting with jaundice, ascites and bone marrow fibrosis

KIM VAIPHEI[1], VIRINDER SINGH[2] & SUBHASH VARMA[3]

*Departments of [1]Histopathology, [2]Hepatology and [3]Internal Medicine, Post Graduate Institute of Medical Education and Research, Chandigarh, India*

**Abstract**
A middle aged chronic alcoholic presented with deep jaundice, markedly enlarged and tender spleen with leukoerythroblastic blood picture and bone marrow biopsy showing mild fibrosis. He was tested negative for HIV, hepatitis B and C viruses. Besides very high serum bilirubin, alkaline phosphatase was raised four times the normal value. Contrast enhanced CT showed enlarged spleen and liver with multiple heterogenous lesions in spleen and tiny hypo-dense lesions in liver. In hospital, he developed haemolytic uraemic syndrome and succumed to his illness. At autopsy spleen weighed 5200 gms and variegated in appearance due to large areas of necrosis and whitish tumour nodules. Histology revealed morphology of an angiosarcoma. Liver was also infiltrated by the tumour mainly in and around portal tract areas.

**Key Words:** *angiosarcoma, spleen, liver, infarcts, jaundice, alcoholic, bone marrow fibrosis, leucoerythroblastic blood, tumour lysis syndrome*

### Case report

A 48-year-old man, alcoholic for 15 years, presented with left upper abdominal mass of 3 months duration, followed by weight lost, jaundice and anorexia. He gave a history of jaundice 6 years previously. On examination he was pale and jaundiced. He had a palpable tender spleen extending up to the umbilicus. The liver was 3 cm below the right costal margin. He had anaemia, lymphocytosis, and low platelet count with raised ESR. His peripheral blood smear showed macrocytes, microcytes and ovalocytes. Bone marrow biopsy showed erythroid hyperplasia, moderate megaloblastosis, dys-erythropoiesis and early myelofibrosis. He had a deranged coagulation profile and raised bilirubin of 25.2 g (conjugated = 16.1 g) per 100 ml. Transaminases and serum alkaline phosphatase were increased to 4-fold. Serum urea, creatinine, uric acid and phosphate levels were increased with low calcium. Serum proteins were reduced with normal albumin globulin ratio. Ascitic fluid analysis showed many neutrophils with no malignant cells, sugar of 86 g and protein of 2.7 g per 100 ml. Blood and ascitic fluid cultures were sterile. Serology for malarial parasite, HIV, hepatitis B and C were negative. Upper GI endoscopy showed grade I oesophageal varices. CECT abdomen showed a large spleen with multiple heterogeneous lesions and enlarged liver with multiple hypo-dense tiny lesions (Fig. 1). Fine needle aspiration of spleen and liver contained necrotic material and haemopoietic cells. Differential diagnoses were: (i) alcoholic cirrhosis with portal hypertension and superadded hepatocellular carcinoma; (ii) disseminated tuberculosis with pre-dominant liver involvement; (iii) idiopathic myelofibrosis.

He was managed with iron, folic acid, vitamin $B_{12}$, and was given fluid challenge, diuretics and steroids for tumour lysis syndrome. Renal function improved transiently with rising potassium level. He developed a breathing problem and had cardio-respiratory arrest.

At autopsy, the spleen weighed 5200 g with thickened capsule. The cut section had a variegated appearance due to whitish tumour deposits and infarcts with a patent hilar vessels. Histology showed a cellular tumour arranged in fascicles with many vascular spaces. Tumour cells were oval to spindle having pleomorphic nuclei with pale cytoplasm,

Correspondence to: Dr. Kim Vaiphei, No. 127/C, Sector-24/A, Chandigarh, Pin-160023, India. Fax: +91-172-744401; E-mail: Kimv@glide.net.in

1357-714X print/1369–1643 online © 2003 Taylor & Francis Ltd
DOI: 10.1080/13577140310001644814

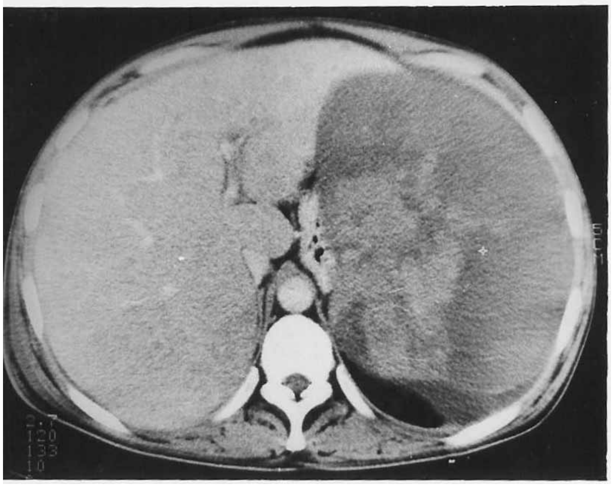

**Fig. 1.** *Contrast CT abdomen showing markedly enlarged spleen with many large irregular heterogeneous masses. There are many tiny diffusely scattered hypo-dense lesions within the enlarged liver parenchyma.*

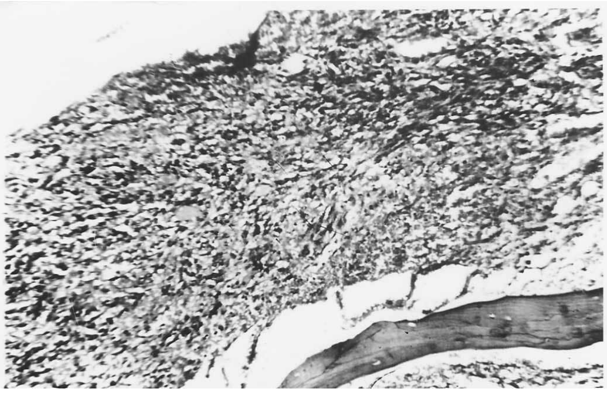

**Fig. 2.** *Photomicrograph of bone section showing tumour cells replacing the marrow (H&E, ×240).*

frequent mitosis intermixed with aggregates of haemopoietic cells and haemosiderin-laden macrophages. Vascular spaces showed many micropapillary fronds. The liver weighed 2800 g, and bile stained with many irregular whitish and haemorrhagic areas (~15 mm). Microscopy showed a similar tumour involving portal tracts with many abortive lesions. Hepatocytes showed steatosis and cholestasis, but no features of alcoholic hepatitis. Peripancreatic lymph nodes showed tumour metastasis. Vertebral bone section showed replacement of marrow space by tumour cells (Fig. 2). Immunostaining showed tumour cells positive for CD31, factor VIII-related antigen, vimentin and S-100, confirming a vascular origin. Week cytoplasmic positivity was seen with CD68, cytokeratin and smooth muscle actin.

### Discussion

Unlike the usual presentation for splenic sarcoma as a mass,[1] the index case had unusual additional features, such as: (i) jaundice and ascites, (ii) tumour lysis syndrome; and (iii) leuco-erythroblastic blood picture with marrow fibrosis. A dominant portal tract infiltration resulted in obstruction of biliary and portal systems. Iron deficiency anaemia has been reported,[2] but there is no report of splenic sarcoma presenting with a leucoerythroblastic blood picture. Extensive tumour necrosis resulted in tumour lysis syndrome, which has been reported with haematological malignancy[3] and soft tissue sarcoma.[4] Chronic exposure to arsenic is a factor known to be involved in development of hepatic angiosarcomas.[5] Indigenously manufactured alcohol in India contains significantly high levels of arsenic.[5] Any individual who has consumed such contaminated alcohol for many years would have tissue accumulation of arsenic. This factor possibly played an important role in the development of angiosarcoma in the indexed case. However, further study is required to document such a relationship.

To conclude, we report an unusual clinical presentation of splenic angiosarcoma for the first time.

### References

1. Manco M, Buononato M, Equitani F. Splenic hemangiosarcoma; a case report. *Oncology* 2000; 59: 98–9.
2. Thomas JP, Porcell A, Sagone AL. Splenic angiosarcoma and iron deficiency anemia in a 43-year-old man. *South Afr Med J* 2001; 94: 640–3.
3. Drakos P, Bar-Ziv J, Catane R. Tumour lysis syndrome in non-haematological malignancies. *Am J Clin Oncol* 1994; 17: 502.
4. Khan J, Broadbent VA. Tumour lysis syndrome complicating bone metastasis of abdominal rhabdomysarcoma. *Paediatr Haematol Oncol* 1993; 10: 151–5.
5. Narang AP, Chawla LS, Khurana SB. Levels of arsenic in alcohol related liver disease. *Drug Alcoh Depend* 1987; 19: 177–80.

## References

[PDF_00130] Drakos P., Bar-Ziv J., Catane R. (1994). Tumor lysis syndrome in nonhematologic malignancies. Report of a case and review of the literature.. Am J Clin Oncol.

[PDF_00133] Khan J., Broadbent V. A. (1993). Tumor lysis syndrome complicating treatment of widespread metastatic abdominal rhabdomyosarcoma.. Pediatr Hematol Oncol.

[PDF_00125] Manco M., Buononato M., Equitani F. (2000). Splenic hemangiosarcoma. A case report.. Oncology.

[PDF_00136] Narang A. P., Chawla L. S., Khurana S. B. (1987). Levels of arsenic in alcohol-related liver disease.. Drug Alcohol Depend.

[PDF_00127] Thomas J. P., Porcell A., Sagone A. L. (2001). Splenic angiosarcoma and iron deficiency anemia in a 43-year-old man.. South Med J.

